# Alternative splicing events are prognostic in hepatocellular carcinoma

**DOI:** 10.18632/aging.102085

**Published:** 2019-07-13

**Authors:** Qi-Feng Chen, Wang Li, Peihong Wu, Lujun Shen, Zi-Lin Huang

**Affiliations:** 1 Department of Medical Imaging and Interventional Radiology, Sun Yat-sen University Cancer Center, Guangzhou, Guangdong 510060, P.R. China; 2 State Key Laboratory of Oncology in South China, Guangzhou, Guangdong 510060, P.R. China; 3 Collaborative Innovation Center for Cancer Medicine, Guangzhou, Guangdong 510060, P.R. China

**Keywords:** alternative splicing, hepatocellular carcinoma, prognosis, consensus cluster, TCGA

## Abstract

Alternative splicing events (ASEs) play a role in cancer development and progression. We investigated whether ASEs are prognostic for overall survival (OS) in hepatocellular carcinoma (HCC). RNA sequencing data was obtained for 343 patients included in The Cancer Genome Atlas. Matched splicing event data for these patients was then obtained from the TCGASpliceSeq database, which includes data for seven types of ASEs. Univariate and multivariate Cox regression analysis demonstrated that 3,814 OS-associated splicing events (OS-SEs) were correlated with OS. Prognostic indices were developed based on the most significant OS-SEs. The prognostic index based on all seven types of ASEs (PI-ALL) demonstrated superior efficacy in predicting OS of HCC patients at 2,000 days compared to those based on single ASE types. Patients were stratified into two risk groups (high and low) based on the median prognostic index. Kaplan-Meier survival analysis demonstrated that PI-ALL had the greatest capacity to distinguish between patients with favorable vs. poor outcomes. Finally, univariate Cox regression analysis demonstrated that the expression of 23 splicing factors was correlated with OS-SEs in the HCC cohort. Our data indicate that a prognostic index based on ASEs is prognostic for OS in HCC.

## INTRODUCTION

Alternative splicing (AS) is an important post-transcriptional regulatory mechanism that increases protein diversity [1]. AS of pre-mRNA transcribed from a single gene can generate isoforms with distinct structures and functions [2]. Approximately 95% of the genes in the human genome undergo AS [3]. Aberrant AS can play a role in cancer development and resistance to therapy [2, 4–6]. For example, splicing factor mutations or alterations in expression can result in the activation of oncogenes and signaling pathways that promote tumorigenesis [7–10]. Alterative splicing events (ASEs) could therefore function as diagnostic or prognostic biomarkers in various cancers. Additionally, cancer-specific splice isoforms or splicing factors could be therapeutic targets.

Hepatocellular carcinoma (HCC) mortality rates are increasing worldwide [11]. Although many studies have identified genes that play key roles in HCC development and progression, few studies have focused on the potential roles of ASEs in the pathogenesis of HCC [12]. The availability of RNA sequencing (RNA-seq) data including The Cancer Genome Atlas (TCGA), and the development of databases such as TCGASpliceSeq (https://bioinformatics.mdanderson.org/TCGASpliceSeq/index.jsp), has enabled the analysis of ASEs in various cancers [13].

**Figure 1:**
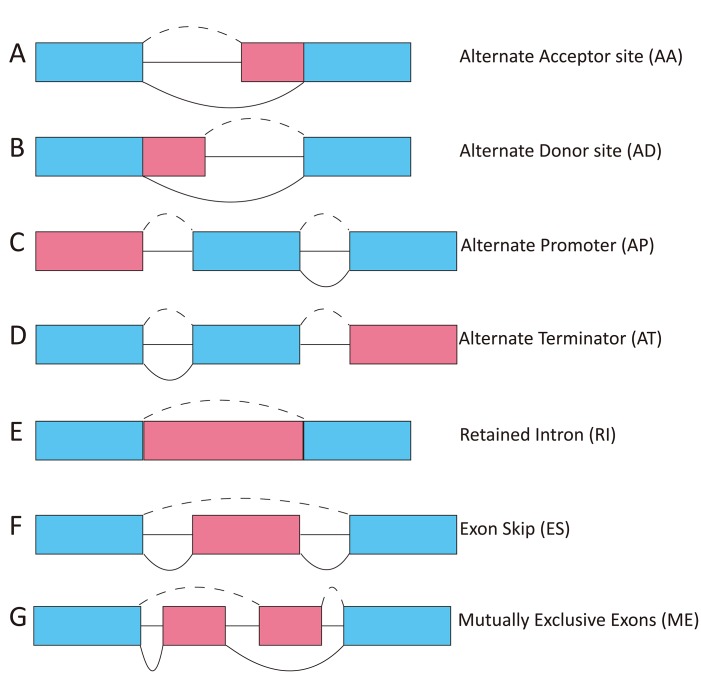
**Diagram showing the seven types of ASEs.** AA, alternate acceptor; AD, alternate donor; AP, alternate promoter; AT, alternate terminator; ES, exon skip; ME, mutually exclusive exons; RI, retained intron.

The TCGASpliceSeq dataset includes seven types of ASEs: (1) exon skip (ES), (2) mutually exclusive exons (ME), (3) retained intron (RI), (4) alternate promoter (AP), (5) alternate terminator (AT), (6) alternate donor site (AD), and (7) alternate acceptor site (AA) (Figure 1) [14, 15]. In this study, we investigated whether the seven types of ASEs were prognostic for overall survival (OS) among 343 HCC patients in the TCGA dataset.

## RESULTS

### Analysis of ASEs in HCC

We analyzed ASEs in pooled mRNA samples from 343 HCC cases included in the TCGA dataset. Individual ASEs were assigned a unique annotation that was a combination of the gene name, splicing type, and the ID number in the SpliceSeq database (AS ID). For example, in the annotation term “C1orf159-AA-20”, the gene name is C1orf159, the splicing pattern is AA, and the AS ID is 20. A total of 34,163 ASEs in 8,985 genes were identified in the cohort of HCC cases: 2,666 AA events in 1,937 genes, 2,331 AD events in 1,663 genes, 6,352 AP events in 2,566 genes, 8,087 AT events in 3,532 genes, 12,327 ES events in 5,343 genes, 137 ME events in 135 genes, and 2,263 RI events in 1,561 genes (Table 1). Thus, individual genes were associated with multiple types of splicing patterns. Additionally, ES was the dominant splicing pattern observed.

**Table 1:** Counts of total and OS-SEs according to ASE type.

**Type**	**Total splicing events**		**OS-SEs**	
	**Splicing events**	**Genes**	**Splicing events**	**Genes**
AA	2,666	1,937	277	257
AD	2,331	1,663	282	248
AP	6,352	2,566	687	381
AT	8,087	3,532	887	486
ES	12,327	5,343	1,423	1,092
ME	137	135	14	14
RI	2,263	1,561	244	219
Total	34,163	8,985	3,814	2,351

### Identification of OS-SEs

**Figure 2:**
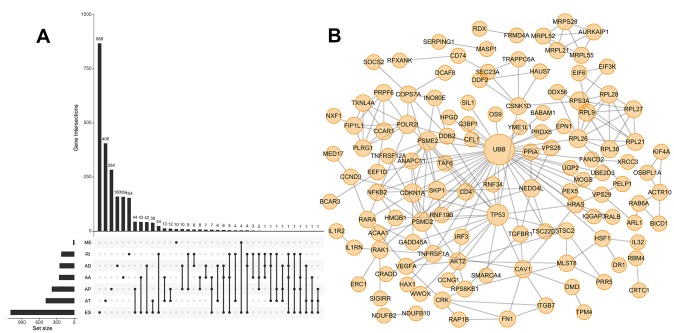
**UpSet plot of OS-SEs and gene interaction network in HCC.** (**A**) UpSet plot showing OS-SEs for HCC; (**B**) Gene interaction network showing all interactions between genes corresponding to the 500 most significant OS-SEs in HCC.

We next performed univariate Cox regression analysis to determine whether ASEs were correlated with the OS of HCC patients. A total of 3,814 OS-SEs were identified, which included ES and AT events in *TP53*, and AA and ES events in *VEGF* (All P < 0.05, Supplementary Table 1). UpSet plots were generated to visualize the interactions between the seven types of ASEs that were associated with OS (Figure 2A). We found that single genes could have multiple OS-SEs. For example, AA, AD, ES, and RI events in *TMEM205*, and AA, AP, ES, and RI events in *CIRBP* were all correlated with OS in HCC patients (Supplementary Figure 1). We selected the 500 most significant OS-SEs and input the genes into Cytoscape to generate gene interaction networks (Figure 2B). Cancer-associated proteins such as P53 and VEGF were found to be major hubs in the resulting networks.

### Prognostic predictors of OS in HCC

**Figure 3:**
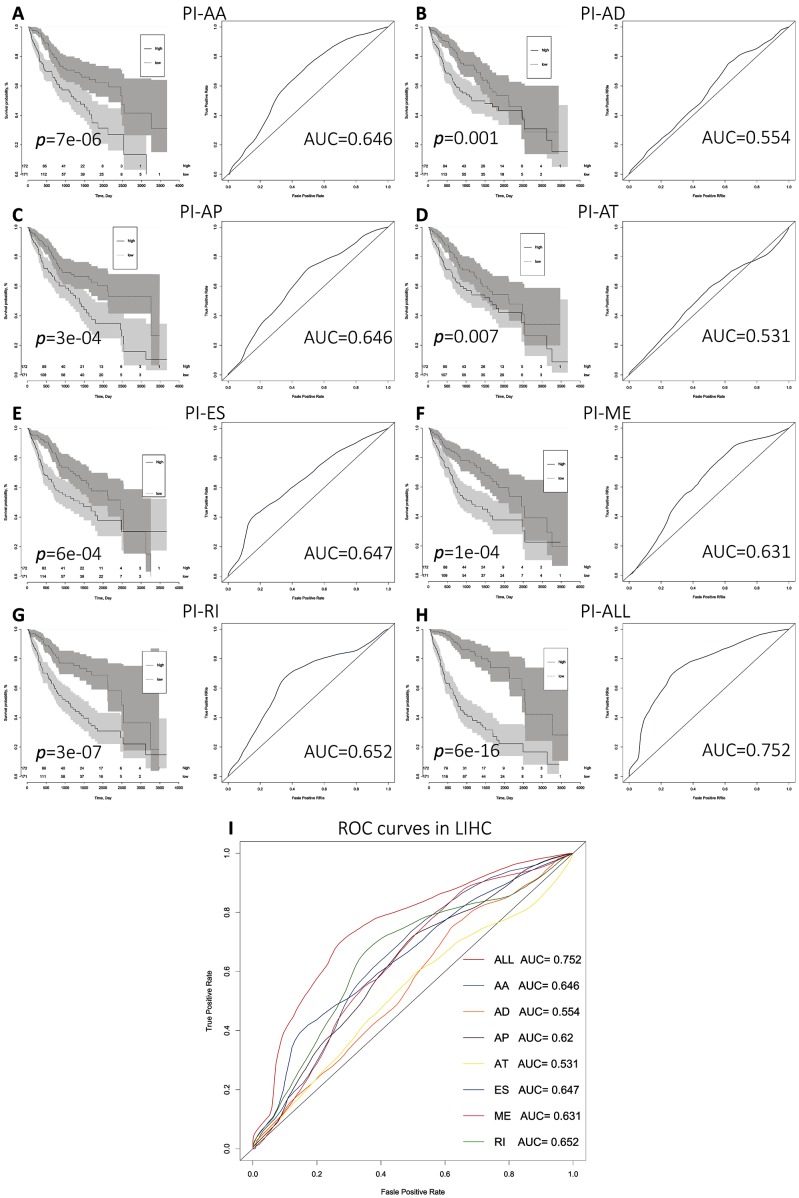
**Comparison of the prognostic efficacy of the eight PIs for OS survival among HCC patients in the low and high risk subgroups.** (**A**–**I**) Kaplan-Meier survival curves for patients in the low and high subgroups for each PI. Time-dependent ROC curves demonstrating the ability of each PI to predict patient survival after 2,000 days.

We next performed multivariate Cox regression analysis based on the 10 most significant OS-SEs for each of the seven splicing types and for all types. Eight prognostic indices (PIs) were generated based on event type: PI-AA, PI-AD, PI-AP, PI-AT, PI-ES, PI-ME, PI-RI, and PI-ALL. The median values for the eight PIs were then used to categorize HCC patients into low and high risk groups. We then analyzed the efficacy of the PIs to predict OS at 2,000 days for the two subgroups using the Kaplan-Meier method. The greatest difference in OS was observed when the HCC patients were stratified based on the median value of PI-ALL (2,542 vs. 768 days in the low and high risk groups, respectively; P = 6e−16) (Figure 3H and Table 2). Receiver operator characteristic (ROC) curves were generated and the area under the ROC curve (AUC) calculated to evaluate the predictive efficiencies of the different models. We found that the PI based on all ASE types demonstrated the greatest efficacy in distinguishing patients with favorable vs. poor prognosis. The AUC for PI-ALL was 0.752, which was significantly higher than those of the other models (Figure 3A–3I).

**Table 2:** Kaplan-Meier survival analysis to evaluate the prognostic efficacy of the different models.

**Type/group**	**Survival, days (95% CI)**	**P-value**
PI-AA		
Low	2,486 (2131 - NA)	7e-06
High	1,271 (899 - 1694)	
PI-AD		
Low	2,116 (1622 - NA)	0.001
High	1,149 (827 - 3258)	
PI-AP		
Low	3,258 (2116 - NA)	3e-04
High	1,372 (1088 - 1791)	
PI-AT		
Low	2,131 (1560 - NA)	0.007
High	1,624 (1005 - NA)	
PI-ES		
Low	2,456 (1624 - NA)	6e-04
High	1,372 (757 - NA)	
PI-ME		
Low	2,456 (1852 - NA)	1e-04
High	1,135 (768 - 2542)	
PI-RI		
Low	2,486 (2131 - NA)	3e-07
High	1,088 (770 - 1622)	
PI-ALL		
Low	2,542 (2456 - NA)	6e-16
High	768 (639 - 1149)	

**Figure 4:**
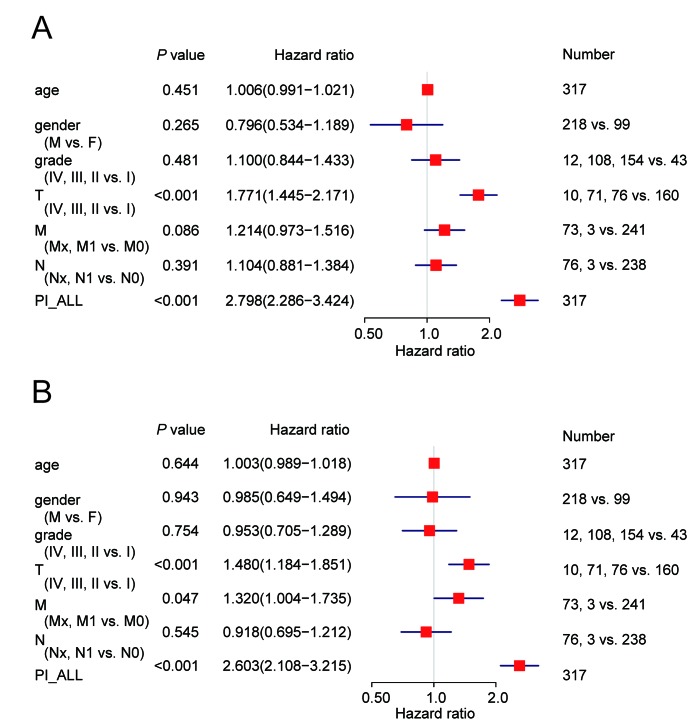
**Cox regression analysis of OS-associated clinical features PI-ALL.** (**A**) Univariate analysis; (**B**) Multivariate analysis.

**Figure 5:**
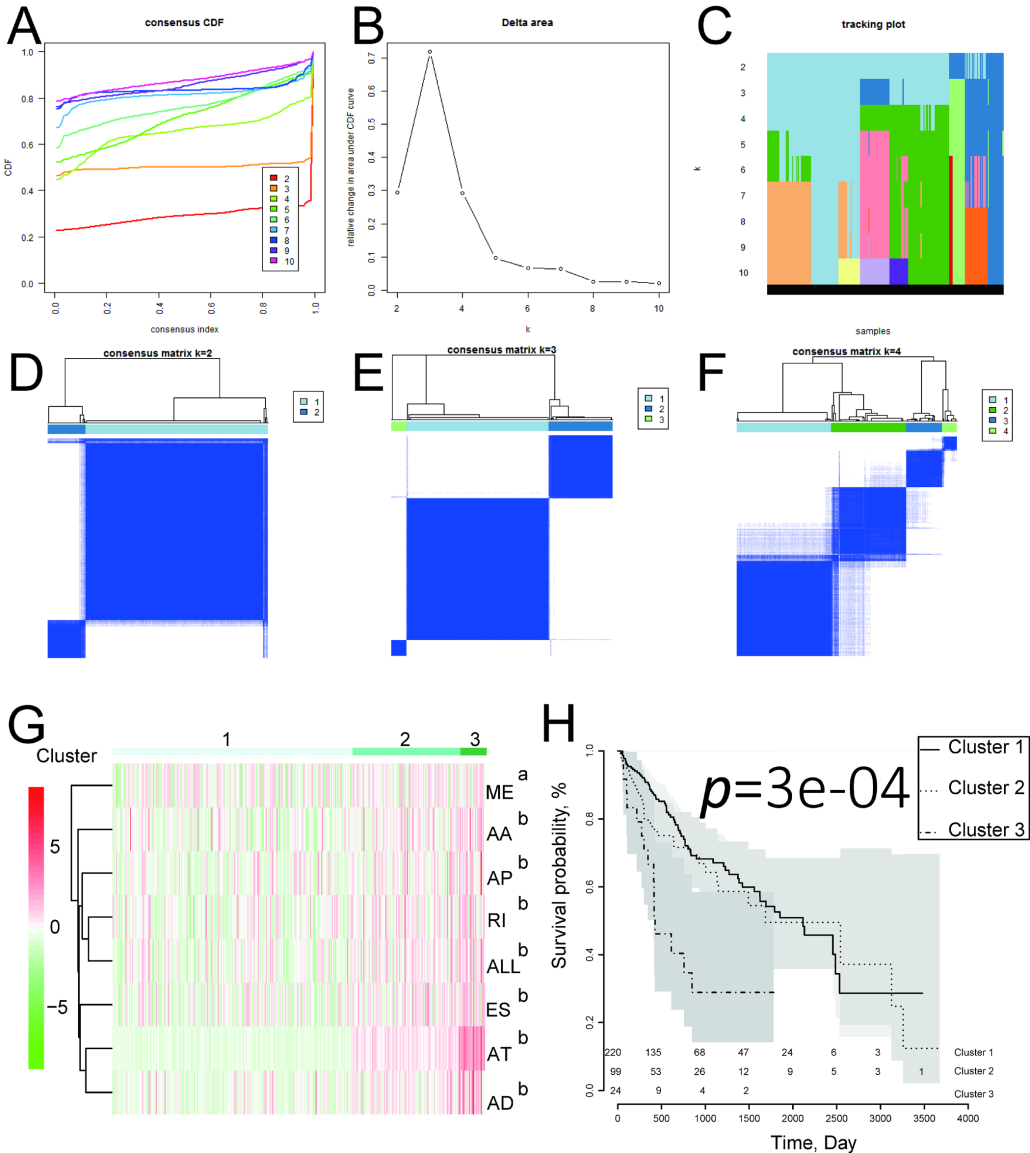
**Identification of three clusters of HCC patients that exhibited distinct ASE features and clinical outcomes using consensus clustering.** (**A**) Cumulative distribution function for k = 2 to 10. (**B**) Relative change in the area under the CDF curve for k = 2 to 10. (**C**) Tracking plot for k = 2 to 10. (**D**–**F**) Consensus clustering matrix for k = 2, 3, and 4. (**G**) Heatmap of the consensus matrix. ^a^P< 0.01, ^b^P< 0.001. (**H**) Kaplan-Meier OS curves for the 343 HCC patients stratified by cluster.

We next performed univariate and multivariate Cox regression analysis to evaluate prognostic value of the PIs and other clinical parameters including age, gender, and tumor stage. The hazard ratios (HRs) for PI-ALL in the univariate and multivariate Cox regression analyses were 2.798 (95% confidence interval [CI]: 2.286–3.424) and 2.603 (95% CI: 2.108–3.215), respectively (Figure 4A–4B). We identified distinct clusters of HCC patients using consensus clustering. We found that k = 3 achieved adequate selection (Figure 5A–5F). Therefore, the patients were clustered into three subgroups. We then compared ASEs and OS among patients in the subgroups (Cluster 1, Cluster 2, and Cluster 3) and found that Cluster 3 had a higher frequency of ASEs compared to Clusters 1 and 2 (ME, P < 0.01; all other patterns P < 0.001; Figure 5G), which was associated with reduced OS and an unfavorable prognosis according to Kaplan-Meier analysis (P = 3e−4, Figure 5H).

### Correlation between OS-SEs and splicing factor expression

**Figure 6:**
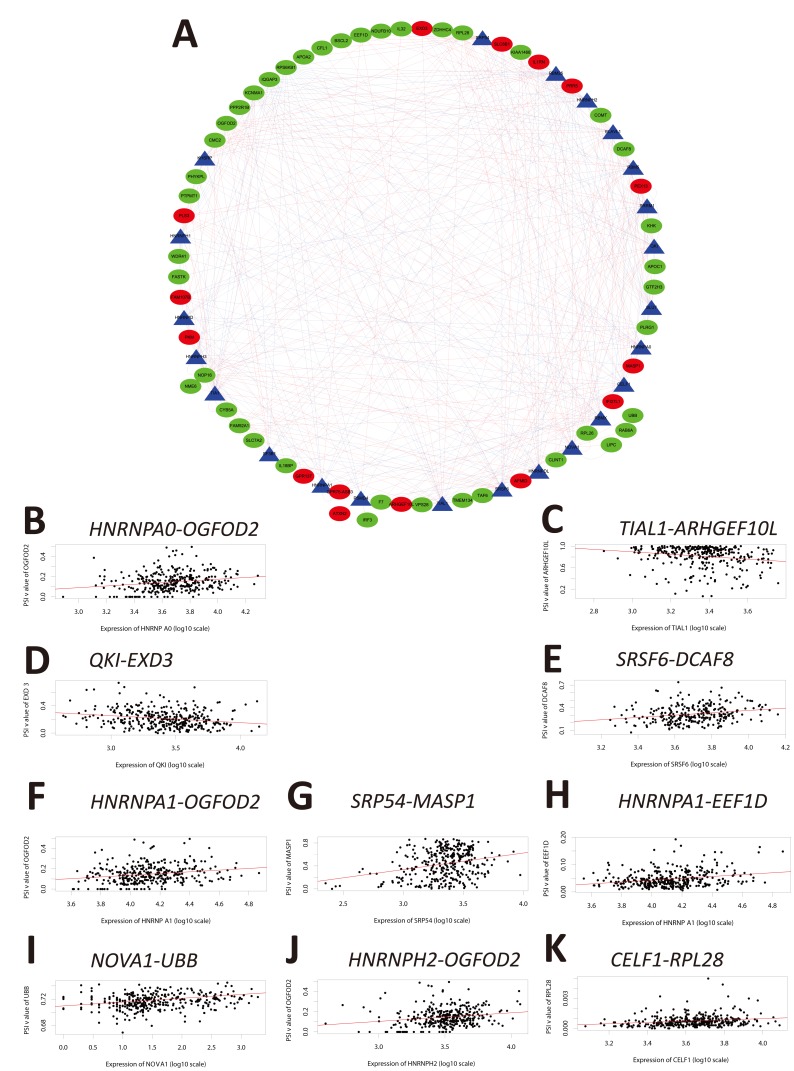
**Correlation analysis between splicing factor expression and OS-SEs.** (**A**) Triangles represent the splicing factors and oval nodes represent the OS-SEs. Red ovals represent the OS-SEs that displayed a positive correlation with OS while the green ovals represent OS-SEs that exhibited a negative correlation with OS. The blue and red lines indicate negative and positive correlations, respectively. (**B**–**K**) Top 10 correlations between the genes corresponding to the OS-SEs and splicing factors according to P-value.

Because alternate splicing is regulated by splicing factors, we investigated whether the OS-SEs were regulated by a subset of splicing factors. Splicing factor expression data were extracted from the SpliceAid2 database (http://www.introni.it/splicing.html). Univariate Cox regression analysis demonstrated that the expression of 23 splicing factors was correlated with OS in the HCC cohort (Table 3). The Kaplan-Meier survival curves are shown in Supplementary Figure 2. We analyzed the associations between OS-associated splicing factors and the percent spliced in (PSI) values for OS-SEs using the Spearman correlation method. Correlation plots were then generated using Cytoscape (Figure 6A and Supplementary Table 2). These results indicated that the expression of 23 survival-associated splicing factors (triangular nodes) was correlated with 447 OS-SEs. Of the 447 OS-SEs, 146 that were associated with favorable OS (red ovals) and 301 were associated with poor OS (green ovals). Interestingly, the majority of the ASEs associated with poor OS were positively correlated with splicing factor expression (red lines), whereas the majority of the ASEs associated with favorable OS were negatively correlated with splicing factor expression (blue lines) (Figure 6A). The 10 most significant associations between genes and splicing factors by P value are shown in Figure 6B–6H. The top splicing factors were HNRNPA0, TIAL1, QKI, SRSF6, HNRNPA1, SRP54, NOVA1, HNRNPH2, and CELF1.

**Table 3:** Survival-associated splicing factors in HCC.

**Gene**	**P value**	**HR**	**Low 95% CI**	**High 95% CI**
*HNRNPH3*	0.000114599	1.000198214	1.000097496	1.000298943
*KHSRP*	0.000140663	1.000101218	1.000049107	1.000153333
*RBMX*	0.000141713	1.000155924	1.000075608	1.000236248
*HNRNPH1*	0.00020603	1.000073002	1.00003445	1.000111554
*SRRM1*	0.000215379	1.000321498	1.000151193	1.000491832
*HNRNPD*	0.000394558	1.000108442	1.000048463	1.000168425
*HNRNPA0*	0.000443747	1.000110171	1.000048696	1.00017165
*SF3B1*	0.000470644	1.000062038	1.000027267	1.000096811
*SRSF6*	0.000693532	1.000123045	1.000051948	1.000194147
*QKI*	0.000735229	1.000133641	1.000056054	1.000211233
*ELAVL1*	0.000759962	1.000221746	1.000092659	1.00035085
*RBM5*	0.00114814	1.000238603	1.000094767	1.000382458
*SLU7*	0.001379709	1.000375697	1.000145488	1.000605959
*RBM25*	0.001517641	1.000235296	1.000089869	1.000380745
*TIA1*	0.00315535	1.000158364	1.000053223	1.000263516
*TIAL1*	0.003611166	1.000261118	1.000085257	1.00043701
*CELF1*	0.005043395	1.000132251	1.000039815	1.000224695
*SRP54*	0.007115521	1.000200907	1.000054596	1.00034724
*HNRNPA1*	0.007692925	1.000023802	1.000006298	1.000041305
*PSMD4*	0.014768251	1.000019396	1.000003803	1.000034989
*HNRNPH2*	0.016730783	1.000114259	1.000020658	1.000207868
*NOVA1*	0.032365794	1.000579807	1.000048732	1.001111164
*HNRNPDL*	0.035203634	1.000072128	1.000005002	1.000139259

### Functional enrichment analysis

**Figure 7:**
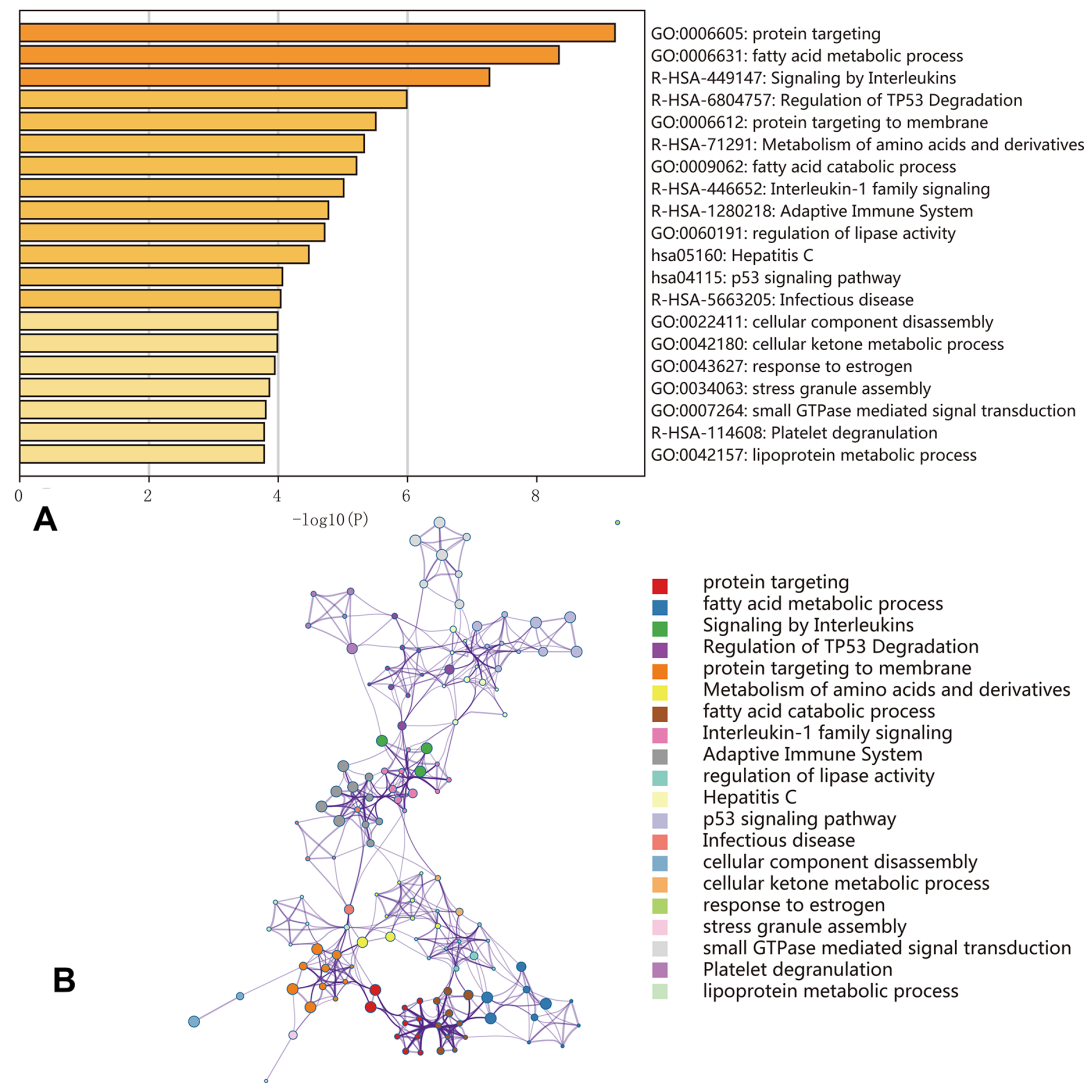
**Enrichment analyses of the genes corresponding to the 500 most significant OS-SEs.** (**A**) Bar graph showing the top 20 results from the enrichment analysis; (**B**) Enrichment analysis showing the gene networks and enrichment of various pathways. Colors correspond to different cluster IDs.

Functional enrichment analysis indicated that genes corresponding to the 500 most significant OS-SEs were involved in ‘protein targeting’, ‘fatty acid metabolic process’, ‘signaling by interleukins’, ‘regulation of *TP53* degradation’, ‘protein targeting to membrane’, ‘metabolism of amino acids and derivatives’, ‘fatty acid catabolic process’, ‘interleukin-1 family signaling’, ‘adaptive immune system’, ‘regulation of lipase activity’, ‘Hepatitis C’, ‘*p53* signaling pathway’, ‘infectious disease’, ‘cellular component disassembly’, ‘cellular ketone metabolic process’, ‘response to estrogen’, ‘stress granule assembly’, ‘small GTPase mediated signal transduction’, ‘platelet degranulation’, and ‘lipoprotein metabolic process’ (Figure 7).

## DISCUSSION

Aberrant AS may play a key role in cancer development [16, 17]. TCGA RNA sequencing data has enabled investigation of AS patterns in various cancers including HCC [18, 19]. For example, Zhu et al. identified an AS signature that was prognostic in HCC using data derived from the TCGA dataset [20]. However, this study included several patients with limited survival and follow-up data. Therefore, we removed them in accordance with more recent studies [14, 21]. Several studies have demonstrated that ASEs are frequently present in HCC tumors [22]. For example, Wu et.al identified 45 ASEs that were observed in tumor tissue from HCC patients but not in adjacent normal tissue samples. These ASEs were associated with survival and cell differentiation [23]. Additionally, Wang et al. demonstrated that a CCDC50S splice variant was modulated by the HBx/SRSF3/14-3-3β complex and promoted tumor progression in HCC through the Ras/Foxo4 signal transduction pathway [24].

In this study, we investigated whether other ASEs could function as prognostic biomarkers in HCC. We identified at least 1,000 distinct ASEs that were observed in HCC (Supplementary Table 1). Functional enrichment analysis revealed enrichment of genes in several pathways that could impact HCC development and progression. The genes corresponding to the ASEs we identified included *TP53* and *VEGF*, which play critical roles in cancer biology. Interestingly, ASEs in the same gene can result in protein isoforms with opposing functional effects. For example, AS of the *BCL2L1* gene results in the generation of two distinct isoforms: BCL-XL and BCL-XS [25]. BCL-XS has pro-apoptotic effects, while BCL-XL has anti-apoptotic effects. The BCL-XL isoform is the predominant variant observed in HCC and protects tumor cells from p53-mediated apoptosis [26]. We identified two ASEs in BCL2, ID_45706 and ID_45707, which were positively and negatively correlated with OS, respectively (Supplementary Table 1). Because these ASEs result in aberrant proteins and were correlated with prognosis, they may play important roles in HCC development.

Alterations in splicing factor expression have been observed in tumor compared to normal tissue [27]. Splicing factors regulate AS and can function as oncogenes or pseudo-oncogenes thereby promoting tumorigenesis [28, 29]. We identified 23 splicing factors that exhibited aberrant expression in HCC tumors (Table 3). Altered expression of several of these factors has been reported previously, such as *QKI* [30], *SRSF6* [7], *HNRNPA1* [31], *NOVA1* [32], and *HNRNPH2* [33]. However, the functions of the majority of the splicing factors we identified in HCC development and progression have not yet been elucidated.

We hypothesized that alternations in splicing factor expression could be correlated with ASEs and OS in HCC. Indeed, the PSI values and network analysis indicated that multiple ASEs were correlated with splicing factor expression in HCC. The majority of the OS-associated splicing factors were highly expressed in HCC and were correlated with poor OS (Figure 6A, Supplementary Table 2). These findings provide insight into the mechanisms by which ASEs function in HCC development and progression. Although our study has several limitations (e.g. sample size, lack of therapeutic strategies, and lack of *in vitro*/*in vivo* functional validation studies), our data indicate that ASEs are frequent in HCC and are correlated with patient prognosis. These ASEs may be part of a prognostic signature in HCC.

## METHODS

### Data extraction

**Figure 8:**
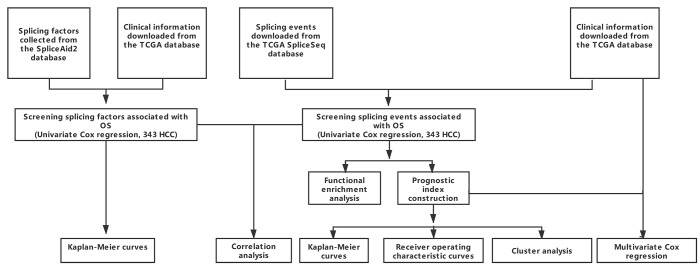
**Overall study design.**

RNA-seq data for 377 HCC cases was downloaded from the TCGA Data Portal (https://tcga-data.nci.nih.gov/tcga/; accessed January 2019). We excluded 28 cases due to limited (< 30 days) of clinical follow-up data. The remaining 349 patients were then matched with their corresponding entries in the TCGASpliceSeq database, and 343 cases were finally enrolled into this study. A schematic of the overall study design is shown in Figure 8.

### Identification of OS-SEs

Univariate Cox regression analysis was performed to identify and analyze OS-SEs. Interactions between the genes corresponding to the OS-SEs were plotted using Cytoscape and the Reactome FI plugin. Metascape (http://metascape.org) was to perform Gene Ontology (GO) term enrichment and the Kyoto Encyclopedia of Genes and Genomes (KEGG) pathway analysis of the genes corresponding to the 500 most significant OS-SEs [34]. The top 20 enrichments were displayed. A P < 0.01 and ≥ 3-fold enrichment were considered significant).

### Analysis of the prognostic values of the PIs

Multivariate Cox regression analysis was performed on the top 10 OS-SEs that had the highest prognostic values for each type of splicing pattern and the top 10 OS-SEs that had the highest prognostic values for all splicing patterns [15]. OS-SEs with P values < 0.05 were selected to construct the PI. The PI was calculated using the following formula β_OS-SE1_ × PSI_OS-SE1_ + β_OS-SE2_ × PSI_OS-SE2_ + · ···· + β_OS-SEn_ × PSI_OS-SEn_, where β corresponded to the regression coefficient. We then evaluated the efficacy of the PIs in predicting cancer status after 2,000 days using ROC analysis with the survivalROC package for R as described [35]. Kaplan-Meier survival curves were generated to analyze the prognostic efficacy of the PIs based on the OS-SEs as described [21]. Finally, Cox regression analysis was performed to calculate the HR values for the PIs and other clinical parameters including T, N, M stage as well as patient age and gender.

### Construction of the correlation network of OS-SEs in HCC

Splicing factor data was extracted from the SpliceAid2 database (http://www.introni.it/splicing.html). Univariate Cox regression analysis was performed to evaluate the correlation between splicing factor expression and OS.

The correlations between splicing factor expression and the PSI values for OS-SEs were analyzed using Spearman’s rank order correlation. Correlation plots were generated using Cytoscape (3.6.0) and the Reactome FI plugin.

### Statistical analysis

R version 3.4.1 was used for all statistical analysis. All P values were two-sided, and a P < 0.05 was considered statistically significant. UpSet was used to visualize the associations between genes and the different types of SEs. Consensus clustering was performed using the ConsensusClusterPlus package for R [36].

## Supplementary Material

Supplementary Figures

Supplementary Table 1

Supplementary Table 2
