# Supplementary material for: Alternative splicing events are prognostic in hepatocellular carcinoma
Source: Aging (Albany NY). 2019 Jul 13;11(13):4720–35. doi: 10.18632/aging.102085 (PMC6660027; doi:10.18632/aging.102085)
Supplement: Supplementary Figures [file aging-11-102085-s003.pdf]

SUPPLEMENTARY FIGURES

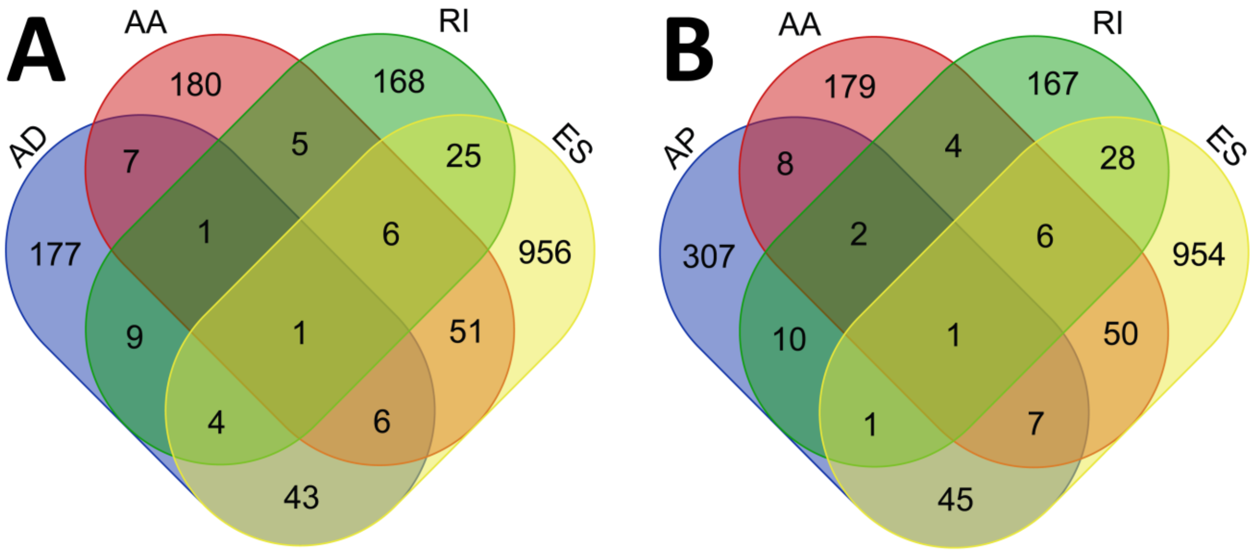

Supplementary Figure 1. Venn diagrams demonstrating that individual genes could be associated with at least four AS patterns. (**A**) AA, AD, ES, and RI events in the *TMEM205* gene; (**B**) AA, AP, ES, and RI events in the *CIRBP* gene.

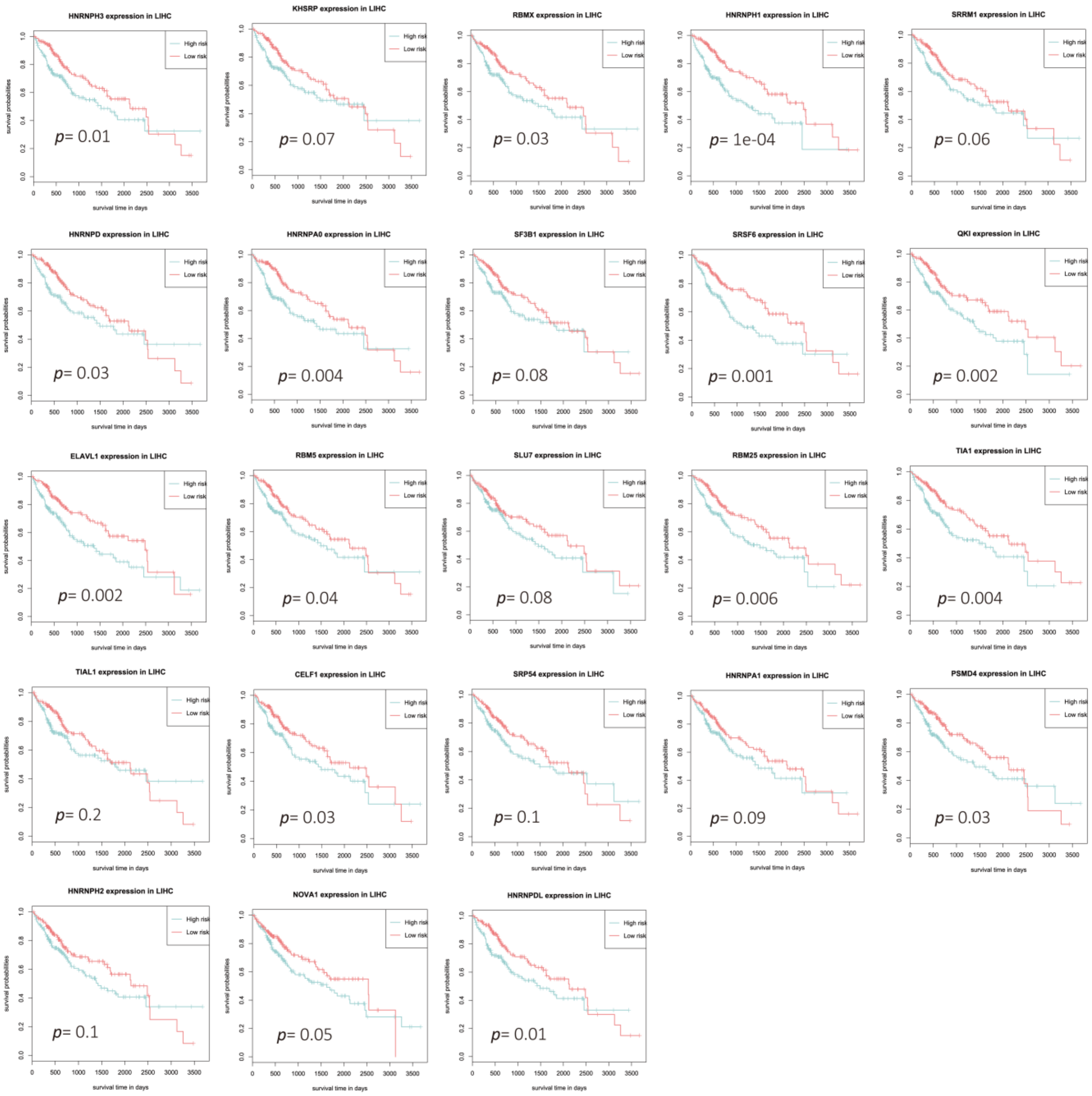

Supplementary Figure 2. Kaplan-Meier curves showing correlations between the expression of 23 splicing factors with OS in the HCC cohort.
